# Demographic, clinical, and pathological features of early onset pancreatic cancer patients

**DOI:** 10.1186/s12876-018-0866-z

**Published:** 2018-09-12

**Authors:** Chara Ntala, Silvana Debernardi, Roger M. Feakins, Tatjana Crnogorac-Jurcevic

**Affiliations:** 1 Barts Cancer Institute, Queen Mary University of London, John Vane Science Centre, Charterhouse Square, London, EC1M 6BQ UK; 2 Department of Cellular Pathology, Royal London Hospital, Barts Health NHS Trust, Pathology and Pharmacy Building, Newark Street, London, E1 2ES UK

**Keywords:** EOPC, Early onset, Pancreatic cancer, Risk factors, Survival

## Abstract

**Background:**

Early onset pancreatic cancer (EOPC), i.e. pancreatic ductal adenocarcinoma (PDAC) occurring in patients below 50 years of age, is rare and there is limited information regarding risk factors, molecular basis and outcome. This study aimed to determine the demographic and clinicopathological features and survival figures for EOPC.

**Methods:**

A retrospective analysis of patients treated at the Royal London Hospital for PDAC between September 2004 and September 2015 was performed. Data on demographics, risk factors, presentation, pathological features, treatment and survival outcome were compared in EOPC and older PDAC patients.

**Results:**

Of 369 PDAC cases identified, 35 (9.5%) were EOPC. Compared to older patients, EOPC patients were more frequently male (71% vs 54%, *p* = 0.043) and less commonly of British origin (37% vs 70%, *p* = 0.002). There was no significant difference regarding the prevalence of any of the risk factors known to be associated with older PDAC patients. Fewer EOPC patients presented with resectable disease (23% vs 44%, *p* = 0.015) and more received adjuvant chemo/radiotherapy (60% vs 46%, *p* = 0.008). The overall median survival and stage specific survival did not differ significantly between the two groups, although a longer survival for localized disease was seen in EOPC patients (25 months (12.9–37, 95%CI) vs 13 months (10.5–15.5 95%CI) for older PDAC patients).

**Conclusions:**

The EOPC patients had different demographics and were more likely than their older PDAC counterparts to be male. Typically they presented with more advanced disease, received more aggressive treatment, and had on overall similar survival outcome.

## Background

Pancreatic ductal adenocarcinoma (PDAC) is the most common pancreatic malignancy. It is the fourth leading cause of cancer death with a 7% five-year survival rate in the United States [1], and is predicted to be the second cause of cancer-related death by 2030 [2]. The mean age of PDAC patients at presentation is 71 years [3]; however, 5–10% of these patients are diagnosed with this malignancy at a young age, when they are less than 50 years old [4–7]. This important subgroup of PDAC patients, often referred to as early onset pancreatic cancer (EOPC), has been poorly studied, and the cause of such an early presentation of the disease remains unknown [6, 8–14].

PDAC is considered to be more frequent in individuals with familial history [15–18] and hereditary genetic syndromes, such as Hereditary Pancreatitis (HP) [19–21], Lynch syndrome [22] and Peutz-Jeghers syndrome [23, 24]. Modifiable risk factors include tobacco exposure, alcohol use, chronic pancreatitis, diabetes mellitus, diet, obesity, previous radiotherapy, as well as certain types of abdominal surgery and infections [25–33]. Also, an inverse association between PDAC and atopic diseases has been identified [34].

In this report, we provide the data of a comprehensive and systematic study on demographics and known PDAC risk factors, as well as detailed clinicopathological, treatment and outcome figures for EOPC patients. The study comprised retrospective examination of notes on PDAC patients collected over the 11-year period between September 2004 and September 2015, from one of the major Hepato-Pancreato-Biliary (HPB) referral hospital centres in London, UK. We compared these data to the data collected for the older PDAC patients from the same centre in order to identify differences and potentially suggest clues to the biology of the disease.

## Methods

### Database search and data extraction

**Fig. 1:**
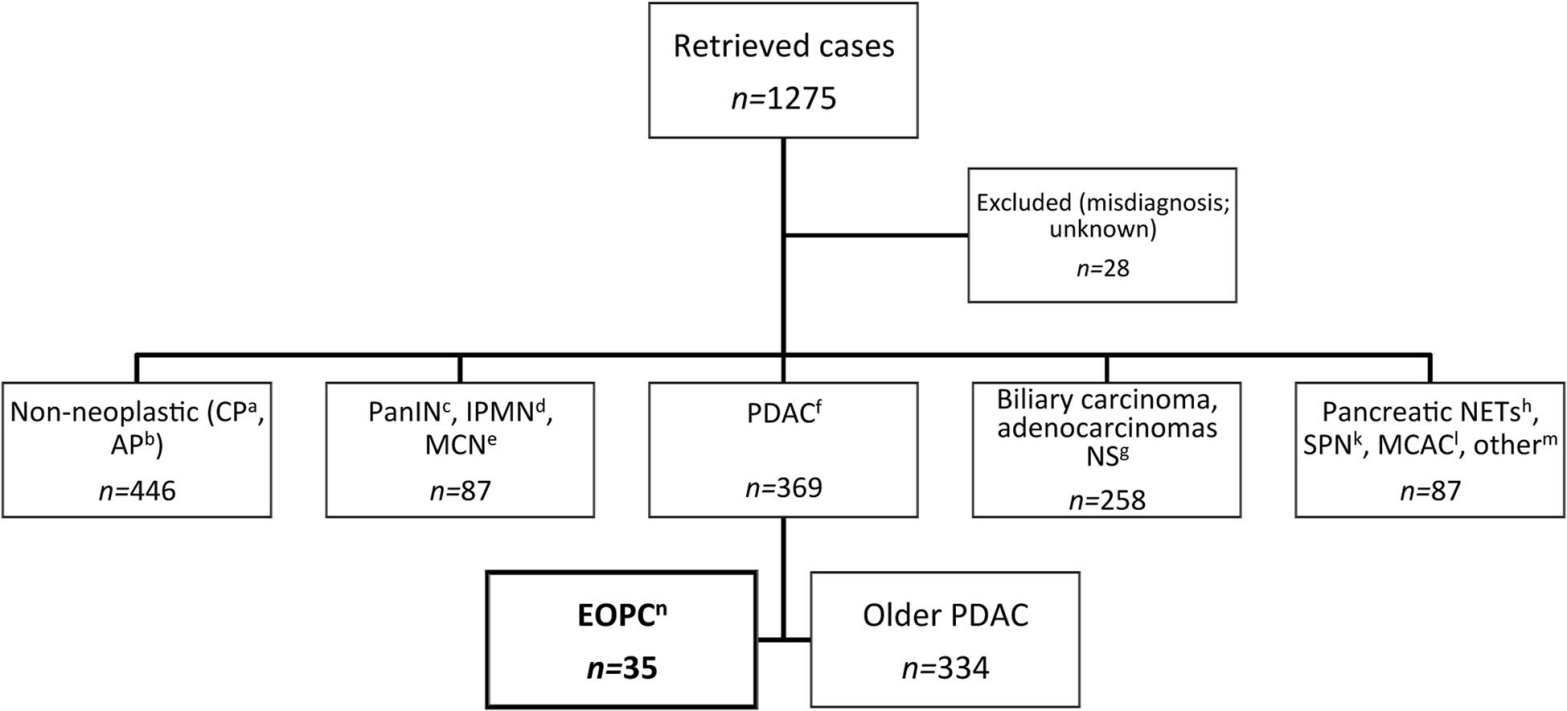
Search Results of London Royal Hospital archives from 18/09/2004 to 18/09/2015. ^a^CP: Chronic Pancreatitis, ^b^AP: Autoimmune Pancreatitis; ^c^PanIN: Pancreatic Intraepithelial Neoplasia; ^d^IPMN: Intraductal Papillary Mucinous Neoplasm; ^e^MCN: Mucinous Cystic Neoplasm; ^f^PDAC: Pancreatic Ductal Adenocarcinoma; ^g^NS: unknown or not specified; ^h^NETs: Neuroendocrine tumours (glucagonoma, large cell neuroendocrine carcinoma, etc); ^k^SPN: Solid Pseudopapillary Neoplasm; ^l^MCAC: Mucinous Cystadenocarcinoma; ^m^other: serous cystadenoma, undifferentiated carcinoma, adenosquamous, signet ring cell carcinoma, mixed ductal-neuroendocrine carcinoma, lymphomas (diffuse large B-cell lymphoma); ^n^EOPC: Early Onset Pancreatic Cancer

We conducted a search of the pathology archives and patients’ database at the Royal London Hospital for period between 18/9/2004 to 18/9/2015 in order to identify patients who were diagnosed with pancreatic ductal adenocarcinoma. We used the topography search codes “pancreatic structure” and “pancreas and duodenum”, and included pancreatic FNAs (fine needle aspirations), core biopsies and surgical resection specimens with a pathology diagnosis of pancreatic ductal adenocarcinoma or adenocarcinoma with features “compatible with”, “suggestive of” or “typical of” pancreatic ductal origin. We excluded all other histologic subtypes and cases of adenocarcinoma of unknown origin, highly suspicious of adenocarcinoma, adenocarcinoma arising from ampulla or bile duct and non-neoplastic diagnoses (Fig. 1). Patients under the age of 50 were grouped into the EOPC cohort and were compared to older patients, referred to here as the older PDAC group. Information extracted consisted of the following: 1) Demographics: age, gender and ethnicity. 2) Epidemiologic and genetic information: history of alcohol excess, obesity (BMI > 30), previous abdominal surgery or radiotherapy, allergy status, and family history of pancreatic cancer or other related malignancies. Specific quantitative data on smoking history in terms of duration, intensity, or recency were rarely available thus smoking history was descriptive (never/ex/current smoker) rather than in accordance with specific definitions (e.g. pack-years). 3) Presenting symptoms: Past medical history of chronic pancreatitis was considered positive if it was present more than 6 months before diagnosis, and recent onset diabetes mellitus was defined as diabetes diagnosed within 3 years before diagnosis. 4) Clinical stage at diagnosis: record followed the American Joint Committee on Cancer (AJCC) and the Union for International Cancer Control (UICC) TNM staging system 7th edition [35]. 5) Surgical management, details of adjuvant and palliative treatment. 6) Survival data. The date of diagnosis was defined as the date when biopsy was taken. The date of last follow-up was used if date of death was not available. The study was done under Research Ethics approval (Reference Number: 05/Q0408/65).

### Statistical analysis

Differences between groups were calculated with two-sided chi-square test (*p* < 0.05 was considered statistically significant). The age was presented as a mean value with ranges, and survival was presented as a median value with 95% confidence intervals (CI). Survival curves were estimated using the Kaplan–Meier method and compared by the log-rank test. All statistical analyses were performed using IBM SPSS Statistics 24.0.

## Results

### Patient demographics, epidemiologic and genetic characteristics

Our search identified 1275 cases; of 369 pancreatic cancer cases 35 were EOPC, comprising 9.5% of the total PDAC population (Fig. 1). The general characteristics of EOPC and older PDAC patients are presented in Table 1. The mean age was 45.71 (33 to 50) years for the young onset population while older PDAC patients had a mean age of 66.19 (51 to 85) years. Gender distribution was significantly different between EOPC and older PDAC groups, with higher rate of male patients in the younger cohort (71% vs 54%, *p* = 0.043). In terms of ethnicity, the majority of older PDAC patients were British (70% vs 37% in EOPC cohort, *p* = 0.002), whereas the EOPC group included a significant proportion of patients from Asian and Central/Eastern European origin. There was no significant difference in terms of modifiable and genetic risk factors between the two groups. First-degree family history in the EOPC cohort is presented in Table 2. Only one of the 35 EOPC patients was affected by a genetic syndrome (Lynch syndrome). Four EOPC (11%) patients and 34 older PDAC patients (10%) had a first-degree relative with history of any type of cancer (*p* = 0.989). None of the EOPC and only 12 (4%) of the older PDAC patients had a first-degree relative with pancreatic cancer (*p* = 0.25). Interestingly, one EOPC patient had two 2nd degree relatives from parental side (an uncle and a cousin) also diagnosed with pancreatic cancer at an early age (50 and 49 years, respectively).

**Table 1:** EOPC and older PDAC demographics, past medical history, environmental and genetic risk factors

Characteristics	EOPC (35), % (≤50 years)	PDAC (334), % (> 50 years)	*p* value
Mean Age	45.71	66.19	
(Range in years)	(33 to 50)	(51 to 85)	
Gender			0.043
Male	25 (71%)	179 (54%)	
Female	10 (29%)	155 (46%)	
Ethnicity			0.002
British	13 (37%)	233 (70%)	
Asian	5 (14%)	9 (3%)	
Black	3 (9%)	22 (7%)	
Other white background	4 (11%)	11 (3%)	
Any other group	2 (6%)	12 (4%)	
Missing	8 (23%)	47 (14%)	
Smoking status			0.850
Current smoker	7 (20%)	55 (16%)	
Ex-smoker	4 (11%)	45 (13%)	
No	13 (37%)	109 (33%)	
Missing	11 (31%)	125 (37%)	
History of alcohol excess			0.556
Yes	9 (26%)	69 (21%)	
No	14 (40%)	140 (42%)	
Missing	12 (34%)	125 (37%)	
Obesity			0.244
Yes	2 (6%)	23 (7%)	
No	8 (23%)	37 (11%)	
Missing	25 (71%)	274 (82%)	
Genetic syndrome			0.989
Yes	1 (3%)	9 (3%)	
No	30 (86%)	266 (80%)	
Missing	4 (11%)	59 (18%)	
History of chronic pancreatitis			0.092
Yes	5 (14%)	96 (29%)	
No	23 (66%)	191 (57%)	
Missing	7 (20%)	47 (14%)	
History of diabetes			0.385
Yes	8 (23%)	58 (17%)	
No	22 (63%)	233 (70%)	
Missing	5 (14%)	43 (13%)	
Allergy^a^			0.853
Yes	2 (6%)	35 (10%)	
No	15 (43%)	228 (68%)	
Missing	18 (51%)	71 (21%)	
Previous radiotherapy			0.838
Yes	1 (3%)	14 (4%)	
No	23 (66%)	261 (78%)	
Missing	11 (31%)	59 (18%)	
Previous abdominal surgery^b^			0.056
Yes	9 (26%)	78 (23%)	
No	9 (26%)	194 (58%)	
Missing	17 (49%)	62 (19%)	

^a^Allergy to penicillin; ^b^Previous abdominal surgery included cholecystectomy, lateral pancreaticojejunostomy

**Table 2:** Family history of cancer in EOPC patients

EOPC patients	Family history of cancer
1	Mother: endometrial cancer
2	Mother: colon and endometrial cancer, mAunt: endometrial cancer, pGrandmother: bowel cancer, pCousin: breast cancer (age 32), Grandmother: stomach cancer^a^
3	Father: esophageal cancer, Brother: colon cancer, Sister: esophageal cancer
4	Sister: lung cancer (age 38)

p: paternal, m: maternal, ^a^Lynch syndrome

### Symptoms at presentation

With regards to symptoms at presentation, obstructive jaundice was the most frequent presenting symptom in both groups, followed by anorexia, abdominal pain, nausea and vomiting. Two EOPC (6%) patients presented with early onset diabetes compared to 40 PDAC (12%) patients (*p* = 0.299).

### Pathology and clinical stage at diagnosis

A significantly lower number of EOPC patients (22.9%) presented with localised resectable disease compared to older PDAC patients (44%) (*p* = 0.015). A similar proportion of EOPC (54%) and older PDAC (42%) patients had locally advanced unresectable disease or presented with distant metastases (23% vs 17%). In EOPC, half of the patients with metastases at diagnosis had a single liver metastasis and the other half had more than one site of metastasis. The most common sites of metastatic disease were liver (75%), lung (37.5%), pleura and omentum (12.5% each). Similarly, in older PDAC patients the most common site of metastasis was liver, occurring alone in just above half of the cases (53.5%) and in combination with other sites in a further 15.5% of tumours. Other single sites were lung (7%), peritoneum (7%), omentum (1.7%) and pleura (1.7%). Tumour grading and AJCC/UICC TNM stage of EOPC and older PDAC patients did not differ, and there was no significant difference in terms of the other pathological characteristics of tumours (location, differentiation, stage, perineural invasion and vascular invasion) between the two groups (Table 3).

**Table 3:** EOPC and older PDAC tumour characteristics and treatment details

Tumour Characteristics	EOPC (*N* = 35), % (≤50 years)	PDAC (*N* = 334), % (> 50 years)	*p* value
Resectable			0.015
Yes	8 (22.9%)	147 (44%)	
No	27 (77.1%)	186 (56%)	
Missing	0	1 (0%)	
Location			0.579
Head/Uncinate	27 (77%)	265 (79%)	
Body/Tail	8 (23%)	62 (19%)	
Missing	0	7 (2%)	
Differentiation grade			0.315
Well	2 (6%)	15 (4%)	
Moderate	16 (46%)	127 (38%)	
Poor	12 (34%)	170 (51%)	
Missing	5 (14%)	22 (7%)	
Stages			0.194
I	2 (5.8%)	16 (5%)	
II*	6 (17.1%)	117(34%)	
III	19 (54.2%)	140 (42%)	
IV	8 (22.9%)	57 (17%)	
Missing	0	4 (1%)	
Perineural invasion			0.348
Yes	12 (34%)	142 (43%)	
No	23 (66%)	192 (57%)	
Perivascular invasion			
Yes	7 (20%)	122 (37%)	0.051
No	28 (80%)	212 (63%)	
Chemotherapy/ radiotherapy			0.008
Yes	21 (60%)	153 (46%)	
No	5 (14%)	131 (39%)	
Missing	9 (26%)	50 (15%)	

*Stage II *p* value = 0.032

### Treatment and survival

**Fig. 2:**
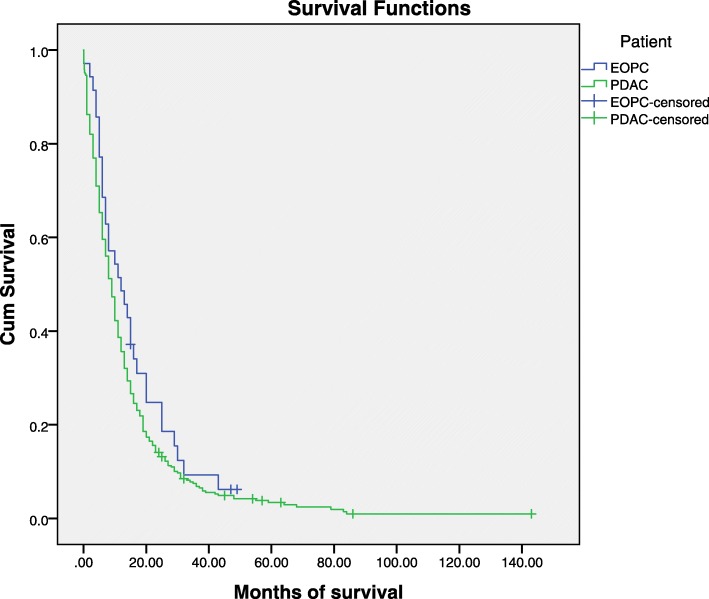
Kaplan Meier overall survival curve in EOPC and older PDAC patients. (y- axis: Cum Survival (cumulative survival), x-axis: time in months)

All 8 (22.9%) EOPC and 147 (44%) PDAC patients with localised disease underwent resection with curative intent. The AJCC/UICC TNM stage of EOPC patients who underwent resection was as follows: IA (*n* = 2), IIA (*n* = 1), IIB (*n* = 5). Sixty per cent of EOPC patients received adjuvant or palliative chemotherapy/radiotherapy whereas only 46% of the older PDAC patients were fit enough to receive the same treatment (*p* = 0.008) (Table 3). Six (17.4%) EOPC and 77 (23%) PDAC patients received a combination of chemotherapy/radiotherapy and surgery with curative intent, out of which one (16.7%) EOPC and six (7.8%) PDAC received this as neoadjuvant treatment that down-staged their disease. The median overall survival of the EOPC patients (12 months, 5–18.9, 95% CI) was higher than in older PDAC patients (9 months, 7.8–10.2, 95% CI) and there was also a trend towards increased stage specific survival (25 vs to 13 months) but neither difference was statistically significant (Fig. 2 and Table 4). Similarly, for patients with locally advanced disease, the median overall survival was 11 months (3.9–18.1, 95%CI) for the EOPC patients and 8 months (6.5–9.4, 95%CI) for the older PDAC patients (*p* = 0.172). Regarding patients with metastatic disease, both cohorts had a median survival of 6 months (*p* = 0.213) (Table 4).

**Table 4:** Overall survival (in months) of EOPC and older PDAC cohorts

	Median survival (months)		
	EOPC (*N* = 35) (≤50 years)	PDAC (*N* = 334) (> 50 years)	*p* value
Entire cohort^a^	12 (5–18.9 95%CI)	9 (7.8–10.2 95%CI)	0.168
Stage I-II	25 (12.9–37 95%CI)	13(10.5–15.5 95%CI)	0.307
Stage III	11 (3.9–18.1 95%CI)	8 (6.5–9.4 95%CI)	0.172
Stage IV	6 (3.2–8.8 95%CI)	6 (4.8–7.2 95%CI)	0.213

^a^Data missing for three EOPC and nine PDAC patients

## Discussion

In this study, we performed a detailed retrospective analysis of EOPC patients with histologically confirmed PDAC in the setting of one of the largest HPB centres in the United Kingdom. While previous EOPC reports also included patients with pathological “variants” (e.g. mucinous cystic neoplasms) which are not included in the current WHO classification of pancreatic ductal adenocarcinoma, our report is based on the most recent PDAC classification and staging. We have used 50 years as the cut-off age for EOPC patients, which, although arbitrary, has been used previously [4–7]. A detailed summary of comparisons between different EOPC studies is shown in Table 5. The main differences between the two PDAC groups were seen in demographic data. EOPC patients in our population were more frequently males, in agreement with previous studies [5–7, 12, 13, 36, 37]. In addition, our EOPC patients were less frequently of white British background and more commonly Asian or from ‘any other white background’. The latter comprised mainly Central and Eastern Europeans. Similar findings were reported by Raimondi et al. who demonstrated in a worldwide study a higher number of male than female patients and a peak in Central and Eastern European countries followed very closely by Asian countries [28]. The association of gender and race/ethnic group with incidence of pancreatic cancer has also been documented in a US report conducted by the National Cancer Institute, where the authors showed the higher incidence and mortality rate in men than in women in each racial/ethnic group, between the age of 30 and 54 [38]. Miller et al., showed up to 50% higher incidence and mortality rate in the black compared to the white population, for the same age group (30–54 years) [38].

**Table 5:** Comprehensive comparison of 14 studies on EOPC, including our study (first column)

	**Ntala C 2018, RLH, UK**	**Piciucchi M 2016, Italy** [7]	**Tingstedt B 2011, Sweden** [5]	**Luttges J 2003, Germany** [8]	**Bergmann F 2006, Germany** [9]	**Duffy A 2009, USA** [10]	**He J 2013, USA** [12]
Length of the study in years	11y (2004–2015)	7y (2006–2013)	15y (1993–2008)	26y (1973–1999)	2006	13y (1995–2008)	34y (1975–2009)
DEMOGRAPHIC							
No. EOPC (%)	35 (9.5%)	25 (8.5%)	33 (5.7%)	10 (2.3%)	7 cases	136 (4.4%)	75 (7.9%)
Age included in study (range)	≤50y (33–50)	≤50y	≤50y (30–50)	≤39 (10-39y)	≤40y (35–40)	≤45y (20–45)	≤45y (31–45)
Male	71% (*p* = 0.043)	68.0%	61.0% (*p* = 0.02)	100.0%	0	54.0%	56.0%
White	37% (*p* = 0.002)						89.0%
Black	9.0%						4.0%
Other	31.0%						7.0%
RISK FACTORS							
Smokers	31.0%	56%* (*p* = 0.001)	73.0%		71.4%	37.0%	37.0%
Use of alcohol	26.0%	36.0%	21.0%				
Obesity (BMI > 30)	6.0%	mean BMI = 27	9.0%				
History of CP	14.0%	0.0%	12.0%		14.3%	13.0%	
History of diabetes	23.0%	4.0% (*p* = 0.00001)	3.0%				
Family history of cancer (1st degree relative)	11.4%	48.0%			71.4%	5.1%	
Family history of pancreatic cancer (1st degree relative)	0.0%	8.0%	3.0%		0	2.2%	
SYMPTOMS AT PRESENTATION							
New onset diabetes	6.0%	4.0%	21.0%				
Weight loss	54.3%	52.0%	55.0%				33.0%
Jaundice	62.8%	16.0% (*p* = 0.006)	61.0%				45.0%
Abdominal pain	45.7%	68.0% (*p* = 0.06)	91.0% (*p* = 0.001^)				32.0% (*p* = 0.06)
PATHOLOGY							
Location-Head	77.0%	64.0% (*p* = 0.03)	79.0%		71.40%		
Location-Body	(B + T) 23%		12.0%				
Location-Tail			9.0%		28.60%		
G1 - Well differentiated	5.7%		20.0%	10.0%	57.2%		
G2 - Moderately differentiated	45.7%		45.0%	45%	42.8%		
G3 - Poorly differentiated	34.3%	61.0%	25.0%	14%			
Perineural invasion	34.3%						64.0%
Localised/Resectable	22.9% (*p* = 0.015)	16.0%	18.0%			25.7%	32.0%
Locally advanced	54.0%	36.0%	27.0% (*p* = 0.005)			20.1%	68.0%
Metastatic	22.9%	48.0%	52.0% (*p* = 0.001)		14.3%	50.0%	
TREATMENT							
Resected cases	22.9% (*p* = 0.015)	16.0%	27.0% (*p* = 0.01)		71.4%	25.7%	(R0) 69.0%
Palliative chemo/radiotherapy	60.0% (*p* = 0.008)	48.0% (*p* = 0.003)	45.0%/36.0% (*p* = ns/0.002)		28.6%		
Supportive treatment	17.1%						
SURVIVAL (months)							
Median OS	12	11	5.7			12.3	19
Median OS (resectable cases)	25					41.8	
Stage associated survival (%; (OS, months))							
Stage I + II	22.8% (25)					25.7% (41.8) (*p* = 0.0001)	I + IIA:32% (27)
Stage III	54.3% (11)	36.0% (11)				20% (15.3)	IIB + III;68% (16)
Stage IV	17.1% (6)	48.0% (7)				50% (7.2)	
Survival rate							
1 year survival rate							
5 years survival rate						3.3%	24.00%
10 years survival rate							17.00%
5 years survival rate I-IIA							42.00%
5 years survival rate IIB-III							16.00%
	**Beeghly-Fadiel A 2016, USA** [6]	**McWilliams RR 2017, USA** [14]	**Ohmoto A 2017, Japan** [37]	**Jiang Q-L 2017, China** [13]	**Lin J-C 2011, China** [4]	**Soliman AS 2002, Egypt** [41]	**Raissouni S 2012, Morocco** [36]
Length of the study in years	25y (1988–2013)	11y (2000–2011)	11y (2002–2013)	16y (1999–2014)	19y (1990–2009)	5y (1995–2000)	5y (2005–2010)
DEMOGRAPHIC							
No. EOPC (%)	118 (8.4%)	226 (11.5%)	17 (1.87%)	156 (8.7%)	25 (10.2%)	165 (22.6%)	32 (17%)
Age included in study (range)	≤49y	≤44y	≤40y (21–40)	≤45y (17–45)	≤49y	≤50y	≤45y (28–45)
Male	60.2%	54.0%	64.7%	75% (*p* = 0.006)			65.0%
White	86.3%						
Black	11.10%						
Other	2.60%						
RISK FACTORS							
Smokers		35% and 6%^§^	58.8%	34.6% (*p* = 0.024)	44.0%		12.5%
Use of alcohol		11%†		35.3%	32.0%		
Obesity (BMI > 30)		19%		(BMI ≥ 28) 16.7%			
History of CP		1.0%			16.0%		
History of diabetes		3.0%		11.5%	4.0%		6.0%
Family history of cancer (1st degree relative)			29.4%	17.3%			
Family history of pancreatic cancer (1st degree relative)	17.5%	8.0%	5.8%	3.8%	8.0%		
SYMPTOMS AT PRESENTATION							
New onset diabetes				11.5%	4.0%		
Weight loss				46.2%	48.0%		43.0%
Jaundice				18.9%	52.0%		68.0%
Abdominal pain				62.1%	72.0%		87.5%
PATHOLOGY							
Location-Head	67.0%		52.90%	69.90%			75.0%
Location-Body	12.3%		(B + T) 47.1%	(B + T) 30%			12.5%
Location-Tail	12.3%						12.5%
G1 - Well differentiated				3.8%%			
G2 - Moderately differentiated				14.7%%			
G3 - Poorly differentiated				26.3%%			
Perineural invasion							
Localised/Resectable	40.0%			28.8%	40.0%		18.7%
Locally advanced	16.0%			34.0%			21.8%
Metastatic	44.0%			37.2%	52.0%		59.3%
TREATMENT							
Resected cases	23.3%		23.20%	20.5%	40.0%	33.1%	18.7%
Palliative chemo/radiotherapy	38.9%		70%	47.4%		30.7%	26.2%
Supportive treatment	37.9%					14.6%	37.5%
SURVIVAL (months)							
Median OS	9.3 (*p* = 0.045)		6.7	8	5.6		6.6
Median OS in resectable			19.6	19	10.3		32
Stage associated survival (%; (OS, months))							
Stage I + II	6% + 34%		23.2% (19.6)	9% (19)			18.8% (32)
Stage III	16.0%		23.5% (18.2)	53.8% (9)			21.8% (7.9)
Stage IV	44.0%		52.9% (5)	37.2% (5)			59% (6.4)
Survival rate							
1 year survival rate			37.5%		28% (40% in resected cases)		
5 years survival rate					4% (10% in resected cases)		
10 years survival rate							
5 years survival rate I-IIA							
5 years survival rate IIB-III							

*Smoking starting age 19.8y (*p* value:0.001); ^ value for matched controls; § % for ≤19 and ≥ 40 packets/year; † > 26 g; C*p* = chronic pancreatitis; B + T = body + tail; y = years; *p* = *p*-value referring to comparison with the older PDAC population (where data available)

One of the putative explanations for the higher incidence of EOPC in males is smoking, a known independent risk factor for pancreatic cancer in all age groups [3, 7, 28, 30, 39, 40]. Indirect association of EOPC and smoking in males was first highlighted by Raimondi et al. who correlated the higher EOPC male/female ratio positively with an early onset of lung cancer (< 50 years of age) [28]. Direct association was provided by Piciucchi et al. [7], who, based on patient interviews, demonstrated a significantly increased risk of EOPC among the ‘current smokers’ group and a positive correlation with the ‘young age at smoking initiation’ [7]. In our cohort, however, only 31% of the total EOPC population had a smoking history which was comparable to the older PDAC cases (29%).

Interestingly, the highest incidence of EOPC patients was reported in two independent studies conducted in North Africa, in Morocco [36] and Egypt [41]: 17% of PDAC patients were younger than 45 years in Morocco [36], and almost 25% in the East Nile Delta were under the age of 50 [41]. In both countries the male to female ratio was around 2:1, in accordance with other reports. Regarding smoking, in the Moroccan population only 12.5% of all EOPC patients were smokers [36]. In contrast, in Egypt smoking is highly prevalent (40% of the general population are smokers). While the authors do not report the smoking history of the EOPC patients, they speculate that occupational and environmental exposure to heavy metals like cadmium, nickel and chromium, as well as other polluting chemicals, could contribute to the high incidence of EOPC in this heavily industrialised region [41]. Unfortunately, we do not have any information on environmental exposure of our study population, and the effect of pollution on our London-based patients would be interesting to explore.

Our study did not identify any differences between EOPC and older PDAC patients in any of the previously identified risk factors for PDAC, i.e. alcohol intake, obesity, history of chronic pancreatitis, history of diabetes, previous abdominal surgery and previous radiotherapy. The rate of recent onset diabetes was somewhat lower in EOPC (6% EOPC vs 12% PDAC, *p* = 0.299) although this was not statistically significant.

None of the patients in our EOPC cohort met the criteria for a familial pancreatic cancer syndrome [42]. Four of the EOPC patients in our cohort had a family history of any cancer but none had a first-degree relative with pancreatic cancer, and only one patient had a hereditary genetic syndrome (Lynch Syndrome) associated with increased risk of pancreatic cancer [22]. A similarly low incidence of familial cases, with no significant difference between young and old PDAC groups, has been reported in other studies. In the cohort described by Duffy et al. [10], only 2.2% of EOPC patients had a family history of pancreatic cancer and no EOPC patients were affected by any of the hereditary syndromes. Tingstedt et al. [5] reported 3% of EOPC with a first-degree relative with pancreatic cancer. However, somewhat higher incidences were found by Piciucchi et al. [7] in both young and older patients, where 8% and 6.3% of cases respectively had a family history of pancreatic cancer. A recent study by Ohmoto et al. [37] suggests a lack of association between hereditary genetic factors and EOPC. The authors assessed the mutation status of 49 genes involved in hereditary syndromes in the germline of EOPC patients younger than 40 years, but did not find any variants [37]. In contrast, James et al. [17] reported an overall incidence of familial pancreatic cancer of 3%, with the percentage of patients ≤50 years of age being significantly higher than among the sporadic cancer patients (36% and 18.3%, respectively, *p* = 0.017). Overall, the underlying factors influencing the young onset of pancreatic cancers remain to be determined.

In our study, the presenting symptoms (obstructive jaundice, abdominal pain, and change in bowel habit, nausea /vomiting, anorexia or weight loss) were largely shared between the two cohorts, and both young and old PDAC patients most commonly presented with obstructive jaundice, probably due to a high incidence of lesions located in the pancreatic head. Interestingly, in a study by Piciucchi et al. [7], jaundice at presentation occurred in only 16% of the EOPC patients, a significantly lower rate than in older PDAC patients (44%, *p* = 0.06) [7]. This was probably due to a lower rate of tumours located in the head of the pancreas (64% vs 83%, *p* = 0.03). Jiang et al. [13] also reported a low frequency of jaundice in EOPC patients, although this could not be explained by tumour location.

The two groups also showed similar pathological characteristics. Poorly differentiated tumours tended to be more common in older PDAC patients (34% vs 51%) and moderately differentiated in EOPC (46% vs 38%), but this was not statistically significant. Furthermore, no difference was observed in the rates of perineural and vascular invasion. Other studies have also showed that the pathological features in EOPC patients are similar to those seen in older PDAC patients, [8, 9] although the presence of more histological variants, especially mucinous carcinomas, has been observed in EOPC cases [8]. Interestingly, a lower rate of KRAS mutations in EOPC patients was found in two studies, [9, 43], although both were performed on a small number of cases (five and seven, respectively).

In our data, EOPC patients presented at a more advanced stage compared to older PDAC group (77% vs 56%, *p* = 0.015) but they were more frequently fit for adjuvant or palliative treatment (60% vs 46%, *p* = 0.008). While the overall survival of EOPC was similar to older PDAC patients with no statistically significant difference, EOPC patients who underwent surgical resection had a longer median overall survival of 25 months compared to 13 months for the same PDAC subpopulation. A similar finding has been observed in previous studies, with the highest survival of 41.8 months for resected cases reported by Duffy et al. [10] which has been attributed to young people having fewer comorbidities and being more suitable candidates for surgery and adjuvant chemotherapy [10, 12, 13, 36, 37]. Furthermore, He et al. also showed that EOPC patients had fewer post-operative complications [12] and McWilliams et al., [14] attributed the better survival rate among young people to a multitude of factors, including race, sex, year of diagnosis, stage of disease, tumour location and treatment [14].

Our study adds to a growing body of literature on the demographic and clinicopathological characteristics of EOPC patients, using contemporary classification and staging manuals. There are some limitations to our study: firstly, the disproportionate sample size of the two comparison groups, EOPC and older PDAC, although reflecting the general incidence of the disease and unavoidable, may have contributed to statistical errors in our analysis. In addition, the retrospective nature of the study has its own pitfalls, which include possible omission of patients that did not have a tissue diagnosis, and review of medical records with sometimes incomplete data. Finally, the study was conducted in a single tertiary expert centre with referred cases and may over-represent the patients that were suitable to undergo tissue biopsy prior to receiving more aggressive treatment. Enlarging the data set through a multi-centre collaborative approach might produce more robust results.

## Conclusions

In conclusion, we present the data from a retrospective study of histologically confirmed PDAC patients over an 11-year period establishing for the first time the demographic and clinicopathological characteristics of EOPC in the multinational PDAC population inhabiting the greater London area. Our results showed demographic differences between EOPC and older PDAC patients, but no difference in association with any currently known risk factors for pancreatic cancer. EOPC patients who undergo surgery have a significantly better survival compared to their older counterparts, which reinforces the value of this therapeutic option, in combination with neoadjuvant chemotherapy/radiotherapy for downstaging of the disease. As risk factors reported previously and in our study do not point to any major differences in demographic and clinicopathological characteristics between EOPC and older PDAC patients, molecular investigations are warranted in order to understand the molecular bases for the occurrence of this highly aggressive malignancy in young populations.

## Abbreviations

AJCC: American Joint Committee on Cancer
BMI: Body mass index
EOPC: Early onset pancreatic cancer
FNA: Fine needle aspiration
HP: Hereditary pancreatitis
HPB: Hepato-Pancreato-Biliary
PDAC: Pancreatic ductal adenocarinoma
PJS: Peutz Jeghers syndrome
TNM: Tumour node metastasis
UICC: Union for International Cancer Control
WHO: World health organisation
